# The Rare Diagnosis of Synchronous Breast and Colonic Cancers: A Case Report and Review of Literature

**DOI:** 10.7759/cureus.13314

**Published:** 2021-02-12

**Authors:** Amira Asaad, Marina Barron, Noreen Rasheed, Philip Idaewor, Abdalla Saad Abdalla Al-Zawi

**Affiliations:** 1 Plastic and Reconstructive Surgery, University College of Medical Sciences, London, GBR; 2 Emergency Department, South West Acute Hospital, Enniskillen, GBR; 3 Breast Radiology, Basildon and Thurrock University Hospital, Basildon, GBR; 4 Pathology, Basildon and Thurrock University Hospital, Basildon, GBR; 5 General and Breast Surgery, Basildon and Thurrock University Hospital, Basildon, GBR; 6 General and Breast Surgery, Anglia Ruskin University, Chelmsford, GBR; 7 General and Breast Surgery, Mid and North Essex University Hospital Group, Basildon, GBR

**Keywords:** multiple primary malignant neoplasms, synchronous tumour, metachronous tumor, breast cancer, colon cancer

## Abstract

Any two or more primary malignant tumors, in which each tumor is not an extension, recurrence, or metastasis of the other lesion, are defined or described as multiple primary malignant neoplasms (MPMN). These tumors are increasingly diagnosed despite their rare occurrence rate. The term synchronous tumors is applied if two different tumors originating in the same patient are detected at the same time or within six months; if the second tumor is detected beyond six months, it is called metachronous. Aetiological factors that may predispose patients to MPMNs have been grouped into three broad categories: familial cancer syndromes and other genetic susceptibility factors, common exposures (e.g. tobacco), and carcinogenic effects of cancer treatment. The likelihood of missing asymptomatic synchronous tumors at the time of diagnosis is due to a lack of definitively set guidelines for synchronous tumors. Studying every individual case may aid us in understanding disease biology, developing diagnostic guidelines, and establishing patient-specific management strategies. We present a case report of synchronous breast and colonic cancer in a female patient.

## Introduction

Multiple primary malignant neoplasms (MPMN) are categorized as synchronous or metachronous and can appear in a single organ or in multiple organs. Synchronous neoplasms are defined as those that occur within six months from the diagnosis of the first primary malignant tumor; metachronous neoplasms are defined as different new neoplasms that appear six months after the first diagnosed tumor [1]. Because of their rarity, synchronous primary cancerous tumors are not easy to diagnose and plan their management. This is because metastatic disease from the diagnosed primary tumor is more frequent than different primaries.

## Case presentation

**Figure 1:**
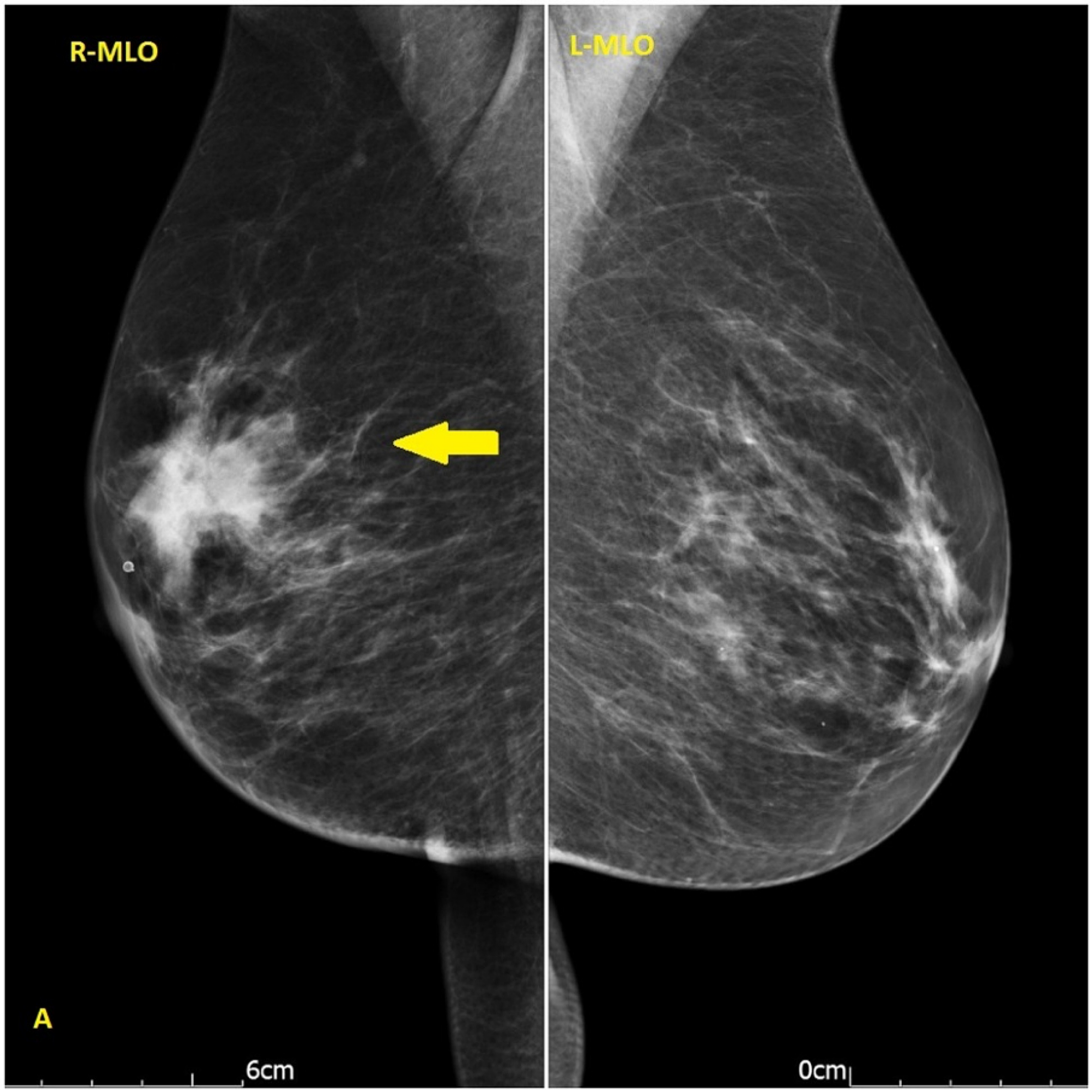
Mammography mediolateral oblique (MLO) view showed a soft tissue density in the right breast with speculated margins measured 42x30 mm (yellow arrow)

**Figure 2:**
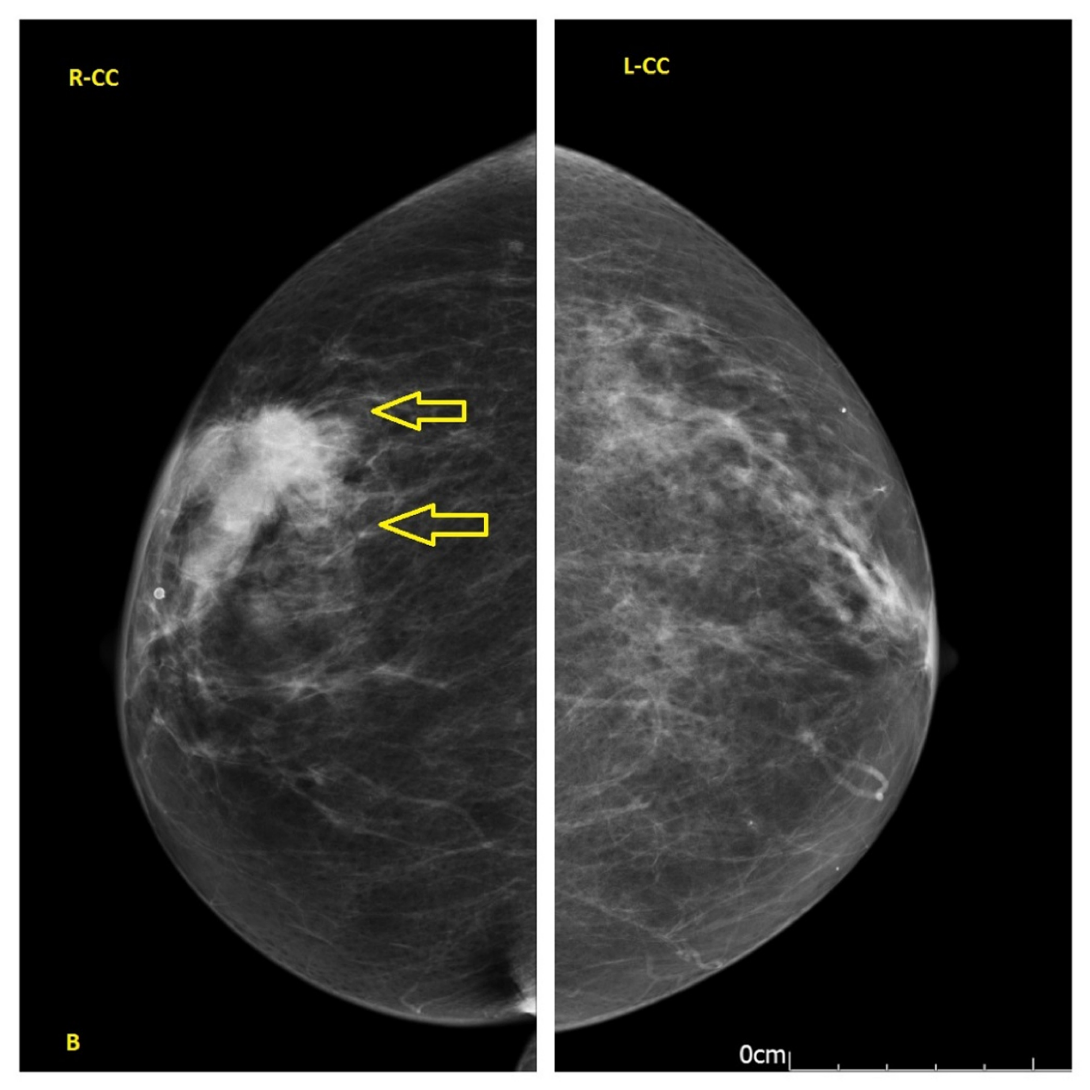
Craniocaudal (CC) view showed a soft tissue density in the right breast with speculated margins measured 42x30 mm (yellow arrow).

**Figure 3:**
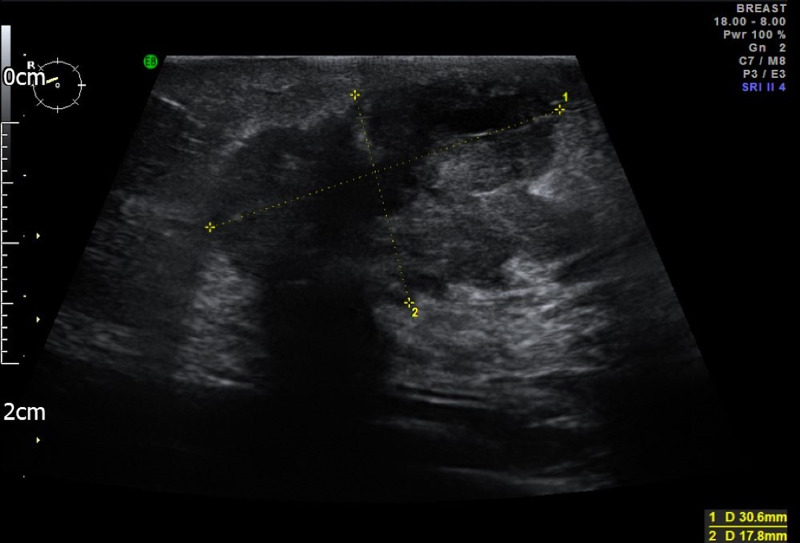
Ultrasound right breast reported a speculated diffuse hypoecoic lesion in the right breast lower outer quadrant with an appearance suggestive of a neoplastic lesion

A 68-year-old female presented with a right breast lump and an inverted nipple. At the same time she was referred to the colorectal surgeons with iron deficiency anaemia. Her past medical history included type II diabetes mellitus as well as hypertension. She had no change in her bowel habit and no blood or mucus in the stool. She had no family history of breast, ovarian, or bowel cancer. On examination, there was an inverted right nipple right with a suspicious lump at 07:00 o’clock position in the right breast. Mammography showed a soft tissue density in the right breast with speculated margins measured 4.2x3 cm (Figures 1, 2). Breast ultrasound scan reported a speculated diffuse hypoechoic lesion in the right breast lower outer quadrant with an appearance suggestive of a neoplastic lesion (Figure 3) in addition to three abnormal-looking nodes in the right axilla.

**Figure 4:**
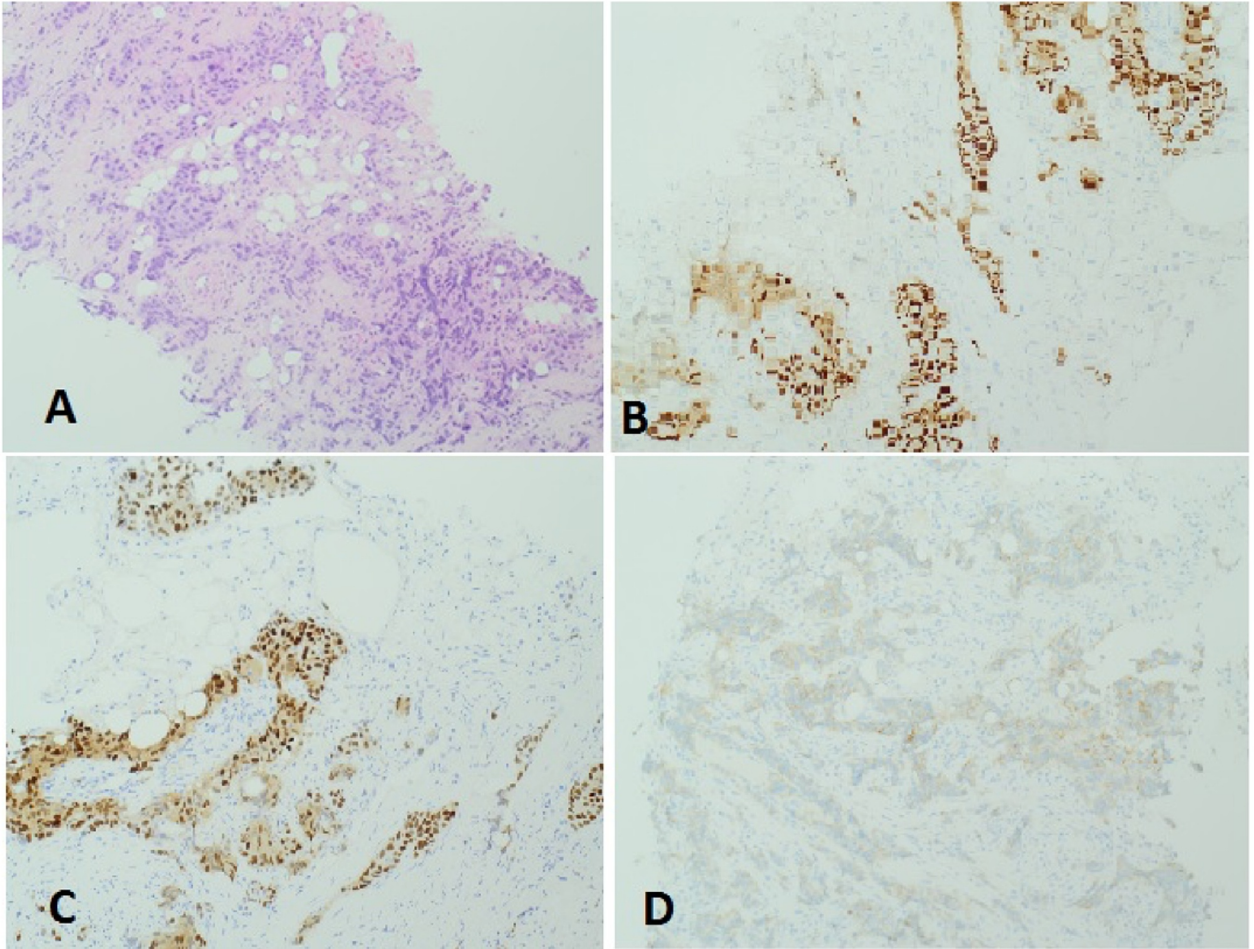
Breast core biopsy; (A) H&E stain showed grade 3 breast invasive ductal carcinoma (B) ER +ve, (C) PR +ve, (D) Her2 –ve. H&E: hematoxylin and eosin, ER: estrogen receptor, PR: progesterone receptor, Her2: human epidermal growth factor receptor 2

**Figure 5:**
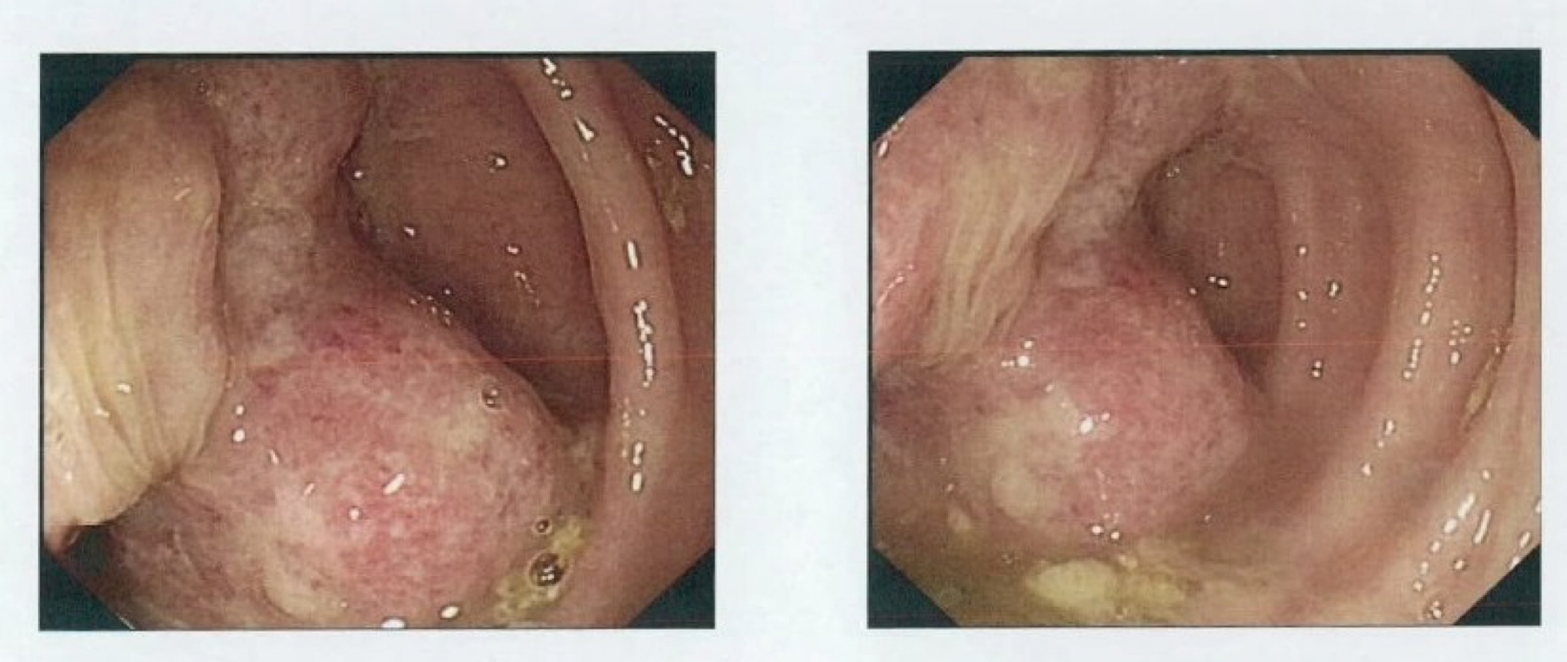
Colonoscopy demonstrating a distal ascending colon malignant looking sessile polyp, 50 mm in diameter.

**Figure 6:**
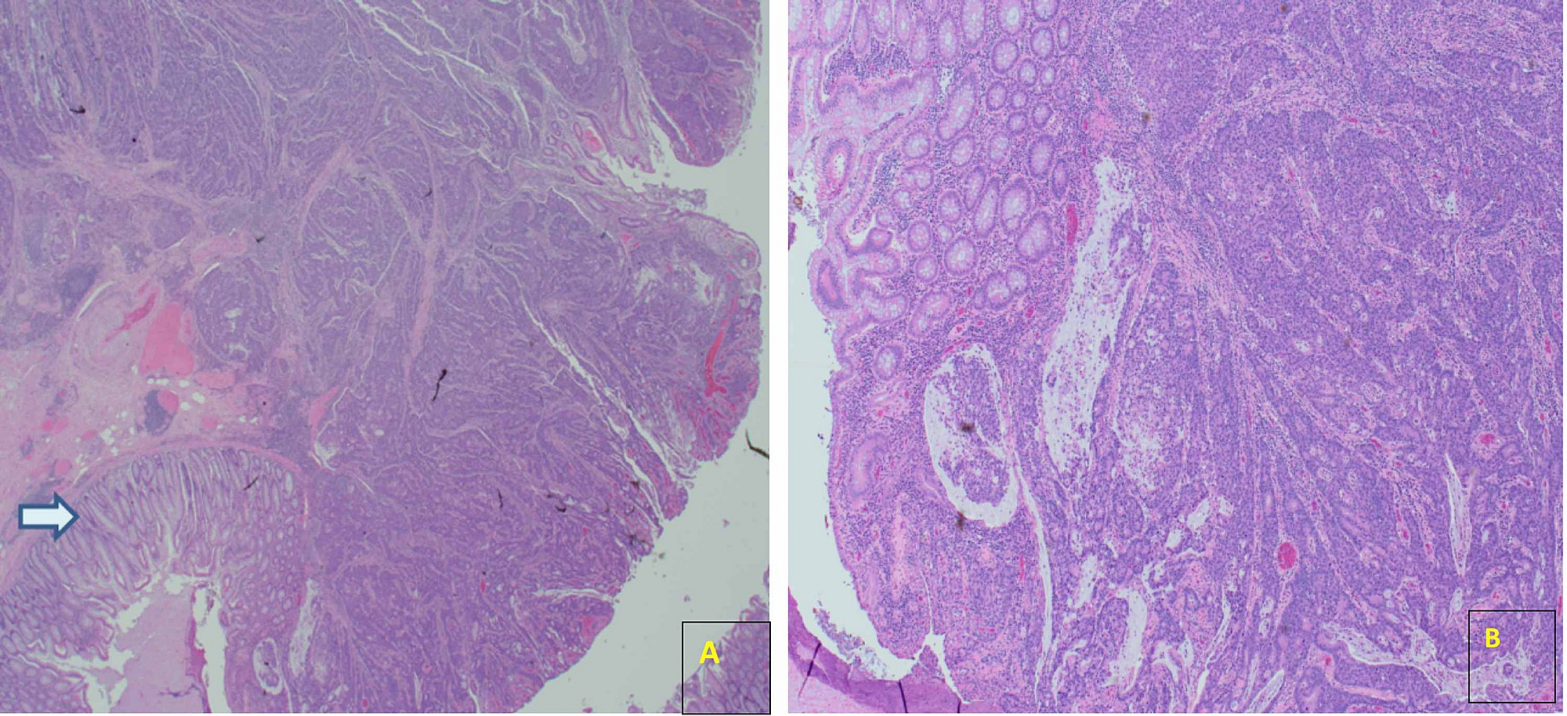
Resection specimen of colon at 1.25 magnification, with adenocarcinoma. Arrow indicates adjacent normal colonic mucosa. B: At higher magnification x4. The adenocarcinoma is moderately differentiated.

**Figure 7:**
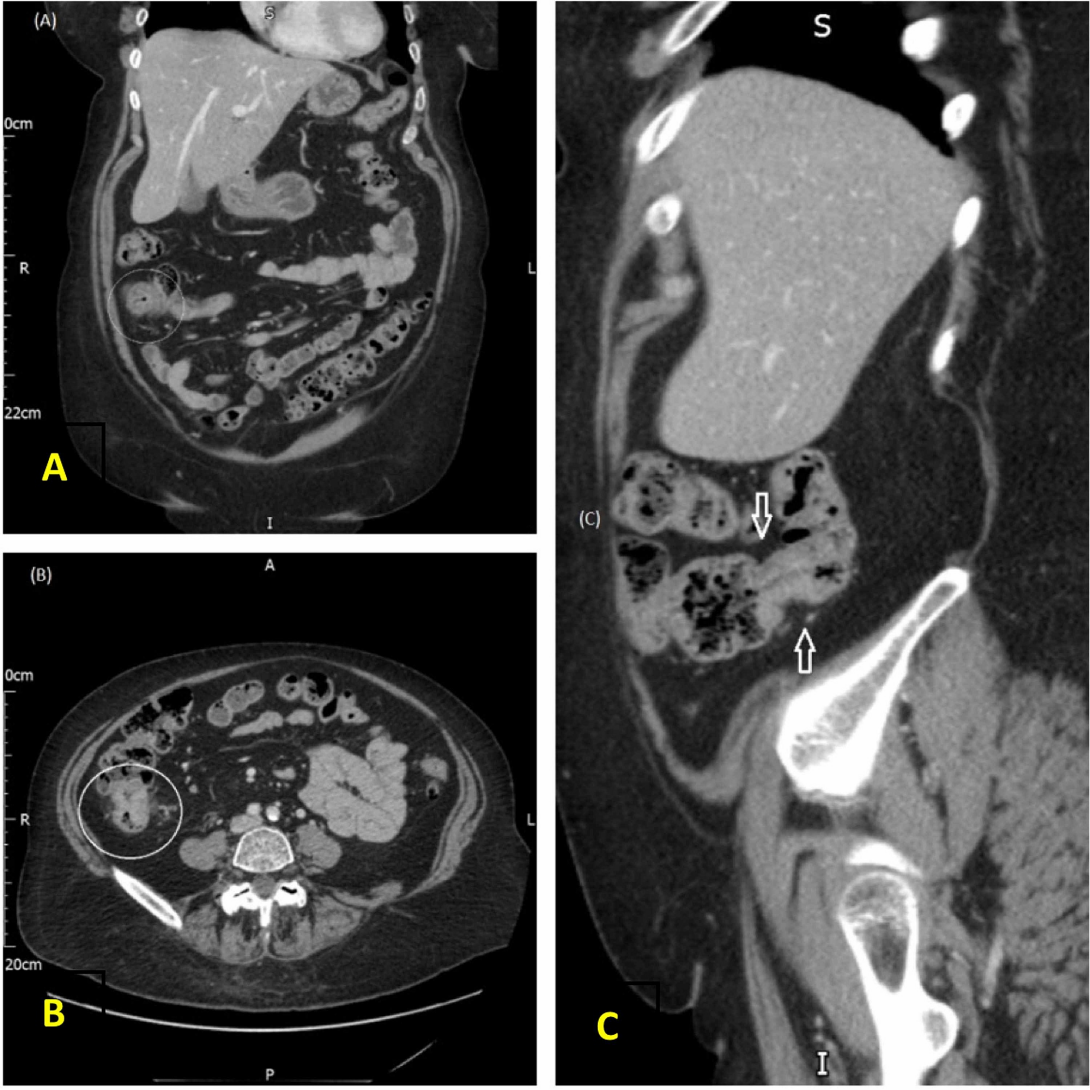
CT abdomen (A) coronal plane, (B) axial plane, (C) sagittal plane, there is a focal spot of wall thickening in the mid ascending colon.

Core biopsy of the lesions revealed grade 3 breast invasive ductal carcinoma with axillary lymph node metastasis (Stage II). Immunohistochemistry showed estrogen receptor (ER) and progesterone receptor (PR) positive, human epidermal growth factor receptor 2 (HER2) negative with Ki-67 more than 40% (Figure 4). Colonoscopy was performed by the colorectal surgeons; it revealed a distal ascending colon malignant looking sessile polyp 50 mm in diameter (Figure 5). The biopsy was consistent with moderately differentiated adenocarcinoma (Figure 6). Immunohistochemistry results were positive for CDX, cytokeratin 20 (CK20), and negative for gross cystic disease fluid protein-15 (GCDFP-15) and GATA-3. This supported the fact that the adenocarcinoma was of a colonic origin. The staging CT scan (chest, abdomen, and pelvis) identified the coexisting neoplasm in the ascending colon; this was seen as a focal spot of wall thickening in the mid ascending colon with few prominent right colic lymph nodes, in addition to sigmoid diverticulosis (Figure 7).

**Figure 8:**
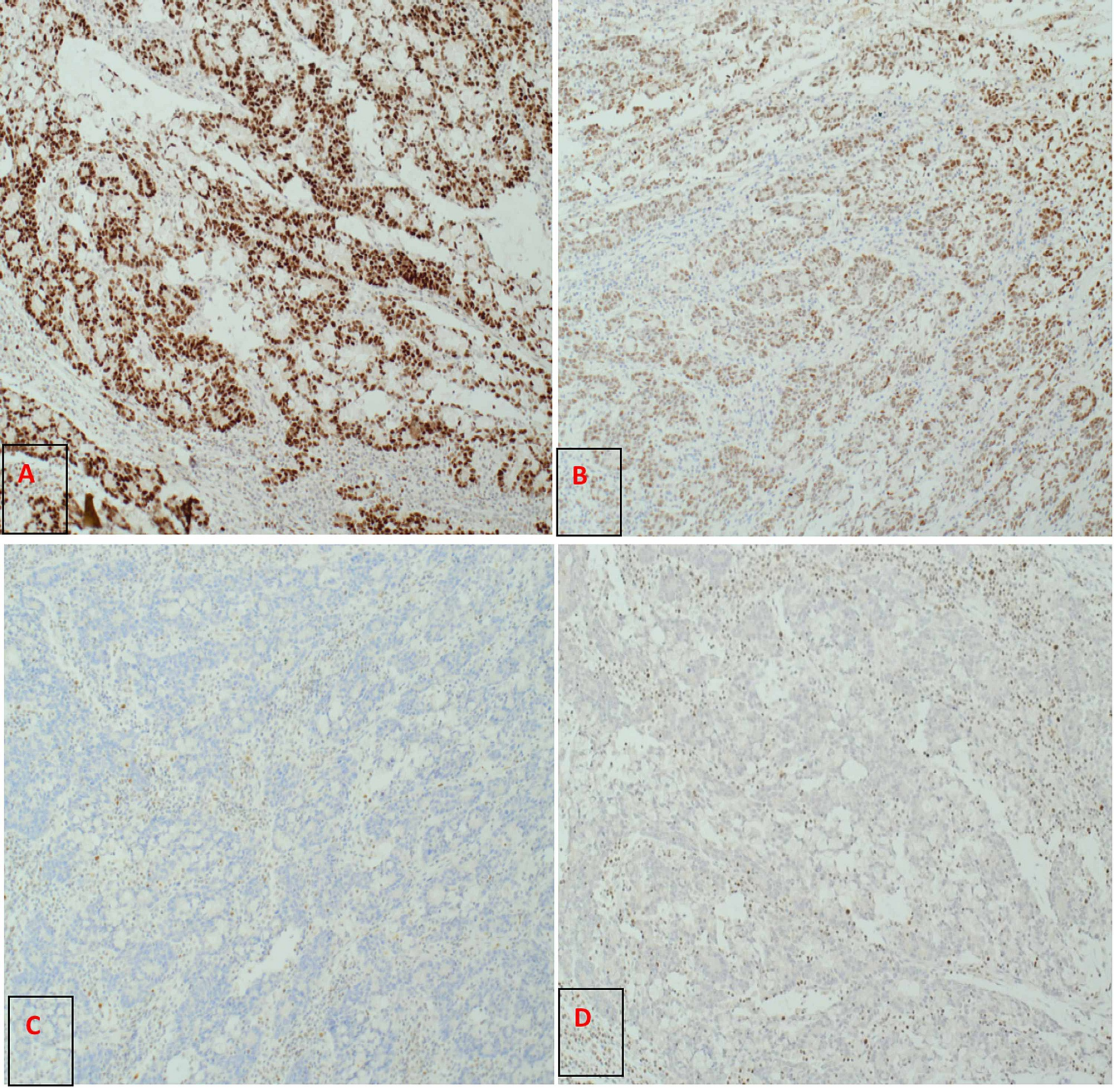
(A) Colonic tumour strongly positive for MSH6 9 (MutS homolog 6 9)x10, (B) week staining for MSH2 (MutS homolog2) x10, (C) MLH1 (MutL homolog 1) is completely negative x10, (D) PMS2 (PMS1 Homolog 2) completely negative x10. Note: The positive – brown cells in the background of (D) are lymphoid cells.

The Breast multidisciplinary team meeting concluded that the patient would require upfront chemotherapy for her breast cancer prior to any surgical intervention for her inflammatory breast cancer. Therefore a decision was made by the colorectal multidisciplinary team meeting to carry out laparoscopic right hemicolectomy to deal with the colonic lesion prior to commencing the chemotherapy. For the breast cancer, she was started on hormonal blockade of letrozole while she was waiting for chemotherapy. The hemicolectomy postoperative histology was consistent with 31 mm moderately differentiated adenocarcinoma, with intramural lymphovascular invasion, completely excised with no disease detected in 16 removed regional lymph nodes, T1, N0, M0; she had an uneventful recovery. The molecular testing revealed that the tumor cells were strongly positive for mutS homolog 6 (MSH6) (Figure 8A) with a week staining for MSH2 protein (Figure 8B). In addition to that, the molecular testing for MutL homolog 1 (MLH1) and PMS1 homolog 2 (PMS2) showed loss of nuclear expression (Figure 8C, 8D). These findings are suggestive of Lynch syndrome (LS). The patient commenced on chemotherapy for breast cancer; after completing the chemotherapy, she underwent mastectomy with axillary node clearance. The post-operative histology revealed no residual invasive carcinoma but 1 mm of ductal carcinoma in situ (DCIS) present; there were two lymph nodes out of the removed nine involved with metastatic cancer. The postoperative plan is to have radiotherapy to the chest wall and supraclavicular fossa, continue with hormonal blockade with letrozole, and commence on bisphosphonates.

## Discussion

The presence of malignancy in two different sites may represent metastatic disease from one primary tumor or the coexistence of two different primaries. Any two or more primary malignant tumors, in which each tumor is not an extension, recurrence, or metastasis of the other lesion, are defined or described as MPMN [1-2]. MPMNs are categorized as synchronous or metachronous and can appear in a single organ or in multiple organs. Synchronous neoplasms are defined as two or more different primary tumors that are detected at the same time or those that occur within six months from the diagnosis of the first primary malignant tumor; metachronous neoplasms are defined as different primary neoplasms that appear six months after the first diagnosed tumor [2]. The risk of developing breast and colonic cancer in the general population is 12% and 16% respectively, the two tumors seldom relate to a single genetic mutation that exposes people to an increased risk of developing cancer. Most colorectal cancers, regardless of the etiology, arise from adenomatous polyps. Although adenomatous polyps are premalignant, only a minority of such lesions transform into cancer. Up to 35% of patients with colorectal cancer have a family history of the disease, suggesting a hereditary predisposition, while only 10% of breast cancer are familial in nature [3]. Different malignancies have been diagnosed in coexistence with breast cancer, such as lymphoma, renal, prostate, esophagus, urinary bladder, uterus, thyroid, colon, melanoma, lung, gastrointestinal stromal tumor (GIST), rhabdomyosarcoma, and ovarian carcinoma [2,4-7].

**Figure 9:**
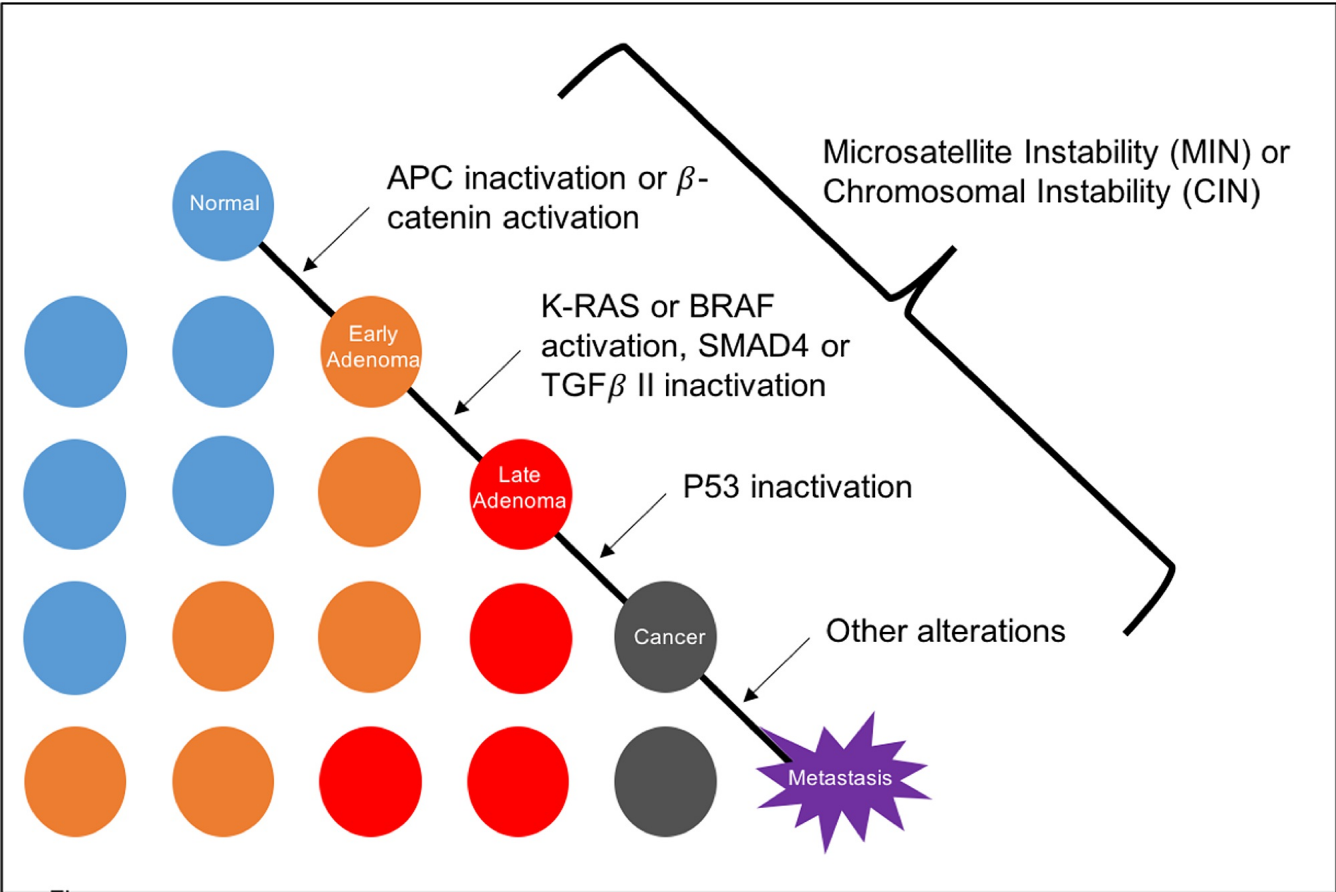
Progressive somatic mutational steps in the development of colon carcinoma. The accumulation of alterations in a number of different genes results in the progression from normal epithelium through adenoma to full-blown carcinoma. Genetic instability (microsatellite or chromosomal) accelerates the progression by increasing likelihood of mutation at each step. Patients with familial polyposis are already one step into this pathway, since they inherit a germline alteration of the APC gene. Adapted from Fauci et al. (2008). TGF, transforming growth factor.

The synchronous bilateral invasive breast cancer is an infrequent clinical entity, and its incidence is reported to be between 0.3% and 12%. The occurrence of breast cancer bi-laterality is encountered more with the invasive lobular carcinoma phenotype, however, there are controversies also about the origin of synchronous cancer (independent primary or metastatic focus) [8]. Colorectal cancer appears with synchronous different primary cancers such as small intestine, ovary, uterus, cervix, mammary gland, kidney, bladder, nasopharynx, lung, stomach, ureter, prostate, oesophagus, thyroid gland, and liver [6,9]. In the inherited cancer disorder, Li-Fraumeni syndrome, which is related to TP53 genetic mutation, patients may encounter early-onset core malignancy as brain tumors, breast cancer, sarcomas, adrenocortical tumors, and leukemia. To a lesser extent patients may develop other tumors as gastric, ovarian, pancreatic, lung and colorectal malignancies [10]. Lynch syndrome (formerly known as hereditary non-polyposis colorectal cancer [HNPCC]) is an autosomal dominant inherited entity of cancer susceptibility. It is related to a germline mutation of one of four genes regulating DNA mismatch repair (MLH1, MSH2, MSH6, and PMS2 [11]); such mutations occur in 12-15% of the colorectal cancers with chromosomal instability [12]. In addition to colorectal cancers, it has been reported that there is an increased risk of having many other types of cancers as a result of this mutation, including gastric, ovarian, biliary, urinary tract, small bowel, brain, and pancreatic cancers [3,13]. Lynch syndrome-associated cancers typically exhibit DNA microsatellite instability (MSI) and loss of mismatch repair (MMR) protein expression [14]. The accumulation of alterations in a number of different genes results in the progression from normal epithelium through adenoma to full-blown carcinoma. Genetic instability (microsatellite or chromosomal) accelerates the progression by increasing the likelihood of mutation at each step (Figure 9) [15]. In our case the colonic tumor specimen, which stained for MLH1 and PMS2, showed loss of nuclear expression; there was weak staining for MSH2, and the tumor cells were strongly positive for MSH6, which is consistent with Lynch syndrome. Some reports mentioned that LS is associated with a higher risk of breast cancer (up to two fold), in particular with MSH6 and PMS2 mutations as well as cases of MSI. The other observation is that age at diagnosis is younger in carrier cases with mismatch repair defects exhibited in the breast tumors [16-18]. Ford et al., in 2012, suggested a wider prospective study to assess if breast cancer is a component of LS as well as to evaluate the prognostic and predictive value of MSI in breast cancer management [16]. It would be reasonable for patients who are LS carriers to be offered adequate screening for breast cancer, given the high probability that most women have their screening mammogram after the age of 50. The immuno-histochemistry panel is crucial to differentiate between the two synchronous primaries.

The immuno-histochemistry panel is a crucial element to differentiate the primary cancers. ER and PR receptors are positive in approximately 70% of breast cancers and Her-2 is expressed in 20% of breast cancer cases; all are used in a routine breast tumor assessment. The Ki-67 proliferation index is increasingly been used as a diagnostic, prognostic, and predictive tool in both breast and colorectal cancer management [19]. GCDFP-15 is a glycoprotein originally detected in the cystic fluid from human cystic mastopathy. It is used as a specific diagnostic marker for neoplasms originating in the mammary tissue, it was negative in the colonic lesion in this case. GATA3, a zinc-binding transcription factor, regulates the precursor cells maturation into breast epithelial cells. It is recognized as a specific and sensitive marker for breast cancer [20].

## Conclusions

The presence of a second malignancy should raise the question of if the tumours different primaries or metastatic disease. Synchronous breast and colon cancers are rare entities and planning an anticancer therapy strategy that deals with more than one primary is a real challenge. However, treatment should be individualised for each case through input from different specialties. It would be reasonable for patients who are LS carriers to be offered adequate breast cancer screening.
